# Antiplatelet Activity of a Newly Synthesized Novel Ruthenium (II): A Potential Role for Akt/JNK Signaling

**DOI:** 10.3390/ijms18050916

**Published:** 2017-04-27

**Authors:** Themmila Khamrang, Kuo-Chen Hung, Chih-Hsuan Hsia, Cheng-Ying Hsieh, Marappan Velusamy, Thanasekaran Jayakumar, Joen-Rong Sheu

**Affiliations:** 1 Department of Chemistry, North Eastern Hill University, Shillong 793022, India; themmilakhamrang@gmail.com (T.K.); mvelusamy@gmail.com (M.V.); 2 Graduate Institute of Medical Sciences, College of Medicine, Taipei Medical University, Taipei 110, Taiwan; hcc4723@gmail.com (K.-C.H.); d119102013@tmu.edu.tw (C.-H.H.); hsiehcy@tmu.edu.tw (C.-Y.H.); 3 Department of Pharmacology, College of Medicine, Taipei Medical University, Taipei 110, Taiwan; 4 Gastroenterologic Surgery Division, Department of Surgery, Yuan’s General Hospital, Kaohsiung 249, Taiwan

**Keywords:** ruthenium complex, platelets, thrombosis, Akt/JNK, ATP, [Ca^2+^]_i_

## Abstract

In oncotherapy, ruthenium complexes are considered as potential alternatives for platinum compounds, and have been proved as promising anticancer drugs with high efficacy and lesser side effects. Platelet activation plays a major role in cancer metastasis and progression. Hence, this study explored the effect of a newly synthesized ruthenium complex, [Ru(η^6^-cymene)(L)Cl]BF_4_(TQ5), where L = 4-phenyl-2-pyridin-2-yl-quinazoline), on human platelet activation. TQ5 (3–5 µM) inhibited concentration-dependent collagen-induced platelet aggregation in washed human platelets. However, this compound only inhibited platelet aggregation at a maximum concentration of 500 and 100 µM against thrombin and 9,11-dideoxy-11α, 9α-epoxymethanoprostaglandin (U46619)-induced stimulation, respectively. TQ5 inhibited collagen-induced ATP release and calcium mobilization ([Ca^2+^]_i_), without inducing cell cytotoxicity. In addition, neither SQ22536, an adenylate cyclase inhibitor, nor 1H-[1,2,4] oxadiazolo [4,3-a]quinoxalin-1-one (ODQ), a guanylate cyclase inhibitor, significantly reversed the TQ5-mediated inhibition of platelet aggregation. TQ5 inhibited the collagen-induced phosphorylation of protein kinase B (Akt) and c-Jun N-terminal kinase (JNK), but did not effectively inhibit extracellular signal-regulated kinase 1/2 (ERK1/2) and p38-mitogen-activated protein kinase (p38-MAPK) in human platelets. Additionally, TQ5 significantly prolonged the closure time in whole blood and increased the occlusion time of thrombotic platelet plug formation in mice. This study demonstrates, for the first time, that a newly synthesized ruthenium complex, TQ5, exhibits potent antiplatelet activity by hindering ATP release and [Ca^2+^]_i_, and by decreasing the activation of Akt/JNK signals. Together, these results suggest that TQ5 could be developed as a therapeutic agent that helps prevent or treat thromboembolic disorders, since it is found to be potently more effective than a well-established antithrombotic aspirin.

## 1. Introduction

Platelets and their activation have been linked to key steps in cancer progression. The influence of platelets on malignancy development has been proposed to be a controlled process that triggers the pathobiology of cancer growth [1]. Cancer cells interact with all major components of the hemostatic system, including platelets. Platelets contribute to critical steps in cancer metastasis, including smoothing tumor cell migration, invasion [2], and arrest within the vasculature [3]. In cellular models of both breast cancer and ovarian cancer, invasiveness has increased following exposure to platelets [2,4]. Platelet contents may be released into the peritumoral space following platelet activation and enhance tumor cell extravasation and metastases [5]. Thrombin has a multifaceted role in hemostasis and represents a key link between primary and secondary coagulation responses. Thrombin has also been linked to tumorigenesis and angiogenesis, with thrombin signaling being a major contributor to metastatic tumor dissemination [6], and it has been detected in situ in numerous tumor types [7]. Thrombin is also the most potent platelet activator; its secretion in human tumor cells activates platelets and recruits them to participate in tumor cell-induced platelet aggregation [8]. A prospective blockade that surrounds the chronic administration of antiplatelet agents in the setting of active malignancy is directly related to the principal role that platelets play in maintaining hemostasis.

The clinical evidence for a higher platelet count, high platelet turnover, and the presence of activated platelets in the circulation generally indicates a poor prognosis in several cancers, such as gastric, colon, and kidney [9,10,11]. A previous study indicated that the obstruction of the GPIIb/IIIa receptor using the monoclonal antibody 10E5, an inhibitor of human platelet GPIIb/IIIa, decreased the lung colonization of cancer cells [12]. An in vivo reduction of pulmonary metastases was found in a murine model of breast cancer by the platelet aggregation inhibitor cilostazol [13]. Wenzel et al. observed decreased ex vivo platelet aggregability and reduced platelet-tumor complex formation while administrating liposomal cilostazol [13]. In addition, Akt is known as protein kinase B, and its three isoforms (Akt1, Akt2, and Akt3) exist in platelets [14]. Studies using Akt inhibitors in human platelets normally support a comparable role of Akt in the stimulation of human platelets [15]. Numerous inhibitors of Akt have been reported to reduce in vitro human platelets aggregation. Besides, it is well-established that JNK and Akt signaling play a vital role in stimulating granule secretion and the integrin α_IIb_β_3_ activation in agonist-induced platelet aggregation and thrombus formation [16]. Therefore, an exploration of the Akt/JNK signaling pathway can disclose some effective targets for drugs which inhibit platelet activation. Despite this, present existing oral antiplatelet agents permanently inhibit their target, making the risk of bleeding more difficult to mitigate. Therefore, it is anticipated that the inhibition of platelet aggregation may be a novel therapeutic target for reducing platelet-tumor composite formation.

Transition metal complexes, including those of ruthenium, have been under exploration for more than a decade, as platforms for producing innovative molecules fostering anticancer properties [17]. These metals exhibit fascinating properties that generate advantages for designing cytotoxic compounds such as: permitting, otherwise distant to carbon-based chemistry, an octahedral geometry, a wide variety of redox potentials, the accessibility of numerous oxidation states (I to IV), and interesting ligand exchange rates, aiding covalent interactions with biological macromolecules [17]. In vitro and in vivo studies have established that numerous ruthenium-based compounds show high cytotoxicity towards a wide range of cancer cells, with reduced side effects [18]. The types of ligand and ancillary arene ligands also play an important role in determining the biological activity of the ruthenium-based organometallic half-sandwich compounds. An interesting study of the structure activity relationship (SAR) for monofunctional Ru^II^ complexes [(η^6^-arene)Ru(Ligand)Cl]^+^, in which the arene is benzene (bz) or a functionalized phenyl ring, including fused ring systems, was reported by Habtemariam et al. [19]. A previous study showed that chemical and structural modifications of nanodiamond surfaces influenced the bioactivity of transported drugs [20]. Instead, we chose to use phenyl substituted quinazoline ligand and *p*-cymene as the ancillary arene ligand in this study, as the Ru(II)-*p*-cymene complex shows a higher ability for DNA and protein binding, and also displays higher cytotoxicity values, than the analogous Ru(II)-benzene complexes. One role of the arene ring in these Ru^II^ arene complexes may be to confer a lipophilic character to the complex, thereby enhancing the uptake into cells. Quinazoline is currently considered a prototypical pharmaceutical lead moiety in medicinal chemistry [21]. Also, the derivatives of quinazoline are currently used as potential anti-cancer, anti-convulsant, and anti-infective drugs [22]. Though there are several in vitro and in vivo biological studies which have shown that ruthenium-based compounds exhibit potential anticancer activity with condensed side effects, no study currently exists that has investigated the effects of ruthenium compounds on platelet aggregation. Based on these hypotheses, a newly synthesized novel ruthenium-based complex, TQ5, has been tested against collagen, thrombin, and U46619-triggered platelet activation in vitro, and the detailed mechanism of the TQ5-mediated inhibition of platelet aggregation has also been characterized.

## 2. Results and Discussion

### 2.1. Results

The quinazoline-based bidentate ligand (L) was synthesized by condensing 2-aminobenzophenone and 2-pyridinecarboxaldehyde in ethanol solution. Mononuclear arene ruthenium complex [Ru(*p*-cymene)(L)Cl]BF_4_ (TQ5) was prepared by treating a slight excess of the ligand with [Ru(*p*-cymene)Cl_2_]_2_, using methanol as the solvent. The product was isolated as tetrafluoroborate salt, producing a good yield. The complex has been isolated as orange colored powder. Based on elemental analysis and electrospray ionization mass spectrometry (ESI-MS), the complex was formulated as [Ru(*p*-cymene)(L)Cl]BF_4_. The ligand and complex were characterized by ^1^H, ^13^C, ESI-MS, and high-resolution mass spectral (HRMS) studies. The ESI-MS data reveal that the complex retains its identity, even in solution.

#### 2.1.1. Nuclear Magnetic Resonance (NMR) Spectroscopy

The new ligand and complex were characterized by NMR spectroscopy. The ^1^H and ^13^CNMR spectra of the ligand in deuterated chloroform (CDCl_3_) and complex indeuterated dimethyl sulfoxide (DMSO-*d_6_*) are displayed in Figures S1–S7 (Supplementary Materials). The spectrum is consistent with the presence of a coordinated *p*-cymene and a bidentate ligand in TQ5. The resonances are assigned based on the available NMR spectral results for the free ligand (L) and that for a closely similar compound [23]. The ^1^H NMR spectrums have a typical pattern which originated from the *p*-cymene moiety. Methyl group singlets appear at 2.26 ppm, multiplets emerge from CH (CH_3_)_2_ groups at 0.92–0.82 ppm, and multiplets from CH (CH_3_)_2_ are seen at 2.4–2.35 ppm. Resonances related to the *p*-cymene ring C–H protons are observed at 6.09–5.3 ppm. Signals due to the quinazoline protons are in the aromatic region from 9.5–7.6 ppm. The ^13^C NMR spectra for the TQ5 complex display resonances attributable to the carbon atoms from *p*-cymene. Methyl group carbons gave signals at 18.3 ppm, signals from the CH (CH_3_)_2_ group methyl carbons occur at 21.6 ppm, and a 30.3 ppm signal is emitted from the CH(CH_3_)_2_ group methylene carbon. Resonances due to aromatic carbon atoms from the *p*-cymene appear at 83.4–104.7 ppm. The signals of the coordinated quinazoline ligand protons have considerably shifted downfield as compared to the free ligand, due to the effect of their coordination with metal centers [24].

#### 2.1.2. TQ5 Inhibits Agonists Induced Platelet Aggregation in Washed Human Platelets

**Figure 1:**
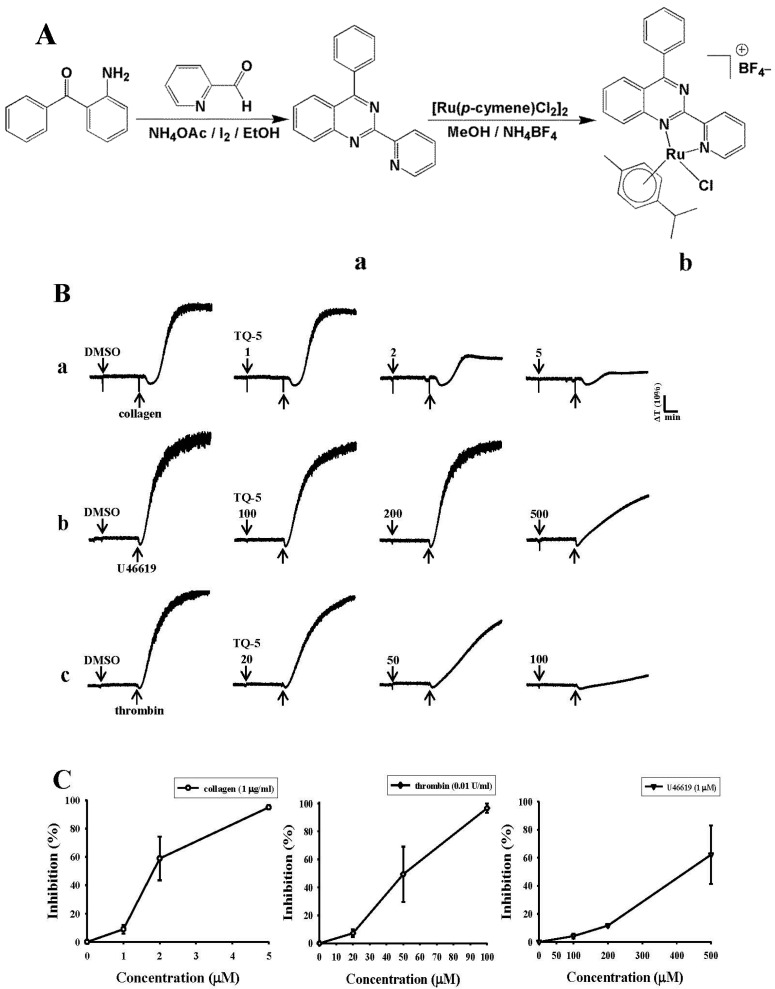
Synthetic method of novel ruthenium complex TQ5 and its ligand 4-phenyl-2-pyridin-2-yl-quinazoline (**A**); Chemical structure of ligand 4-phenyl-2-pyridin-2-yl-quinazoline (**a**) and ruthenium complex, TQ5 (**b**); TQ5 inhibits collagen-induced platelet aggregation (**B**); Washed platelets (3.6 × 10^8^ cells/mL) were incubated with solvent control [(0.5% dimethyl sulfoxide (DMSO)] or TQ5 (1–5 µM for collagen, 100–500 µM for U46619 compound and 20–100 µM for thrombin inducers) for 3 min in an aggregometer cuvette; Then, 1 µg/mL collagen (**a**); 1 µM U46619 (**b**); and 0.01 IU/mL thrombin (**c**) was added to induce platelet aggregation for 6 min; Statistical analysis of three independent experiments (**C**).

TQ5 inhibited platelet aggregation in washed human platelets stimulated by collagen (1 µg/mL) in a concentration-dependent (1–5 µM) manner (Figure 1(Ba)). Although TQ5 did not show a concentration-dependent inhibition on platelet aggregation stimulated by 9,11-dideoxy-11α, 9α-epoxymethano prostaglandin (U46619, 1 µM), and thrombin (0.01 U/mL), it inhibited platelet aggregation in a maximum concentration of 500 and 100 µM, respectively (Figure 1(Bb,Cc)). Moreover, since TQ5 is a newly synthesized ruthenium complex and there are no previous studies, we compared the observed effect of this compound with a well-established antithrombotic aspirin. Using the results, we found that TQ5’s inhibitory effect in collagen (1 µg/mL)-induced platelet aggregation was more potent than that of aspirin (50 µM) (data not shown).

#### 2.1.3. Effect of TQ5 on Adenosine Triphosphate (ATP) Release and [Ca^2+^]_i_ Mobilization in Human Platelets

**Figure 2:**
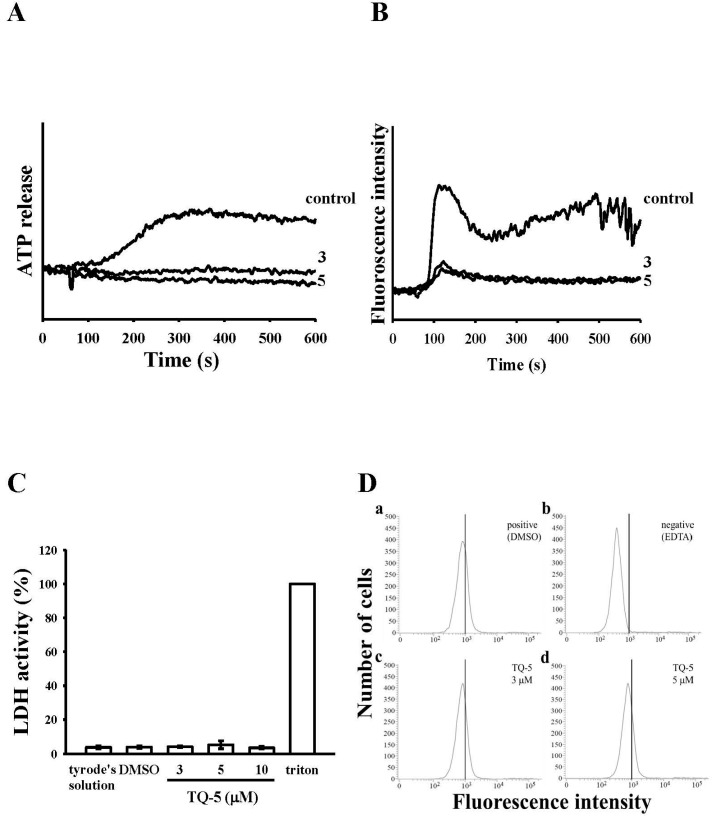
Effects of TQ5 on collagen-induced ATP release, relative [Ca^2+^]_i_ mobilization, cytotoxicity, and on fluorescein isothiocyanate (FITC)-collagen binding in human platelets. Washed human platelets (3.6 × 10^8^ cells/mL) were preincubated with TQ5 or a solvent control (0.5% DMSO) and subsequently treated with 1 µg/mL of collagen to stimulate adenosine triphosphate (ATP) release reaction (**A**); to induce the cytoplasmic influx of Ca^2+^ from intracellular stores (**B**); to induce cytotoxicity (**C**) or to check the direct binding of FITC-collagen (**D**), as described in the materials and methods section. Data are presented as the means ±S.E.M. (*n* = 3).

The result of TQ5 on ATP release and [Ca^2+^]_i_ mobilization in collagen-stimulated human platelets is shown in Figure 2A,B. Collagen (1 µg/mL)-induced ATP release from a dense human platelets was significantly inhibited by TQ5 in a concentration-dependent (3 and 5 µM) manner. In order to study the effect of TQ5 on the [Ca^2+^]_i_ mobilization, the level of [Ca^2+^]_i_ was measured in washed human platelets after treatment with collagen (1 µg/mL). As shown in Figure 2B, the collagen caused a rapid, but transient increase in [Ca^2+^]_i_, and TQ5 (3 and 5 µM) blocked this increase of [Ca^2+^]_i_.

#### 2.1.4. TQ5 Either Not Induced Cytotoxicity or Not Directly Binds to the Platelet α_IIb_β_3_Integrin in Platelets

We examined effects of TQ5 on the cell toxicity by measuring the extracellular activity of lactate dehydrogenase (LDH). LDH is a cytosolic enzyme present in most eukaryotic cells, which is released into the culture medium upon cell death due to the damaged plasma membrane. The LDH study revealed that TQ5 (3–10 µM) incubated with platelets for 20 min did not significantly increase LDH activity in platelets (Figure 2C), indicating that TQ5 does not affect platelet permeability or induce platelet cytolysis. Triflavin is an α_IIb_β_3_ disintegrin that inhibits platelet aggregation by directly interfering with fibrinogen binding to the α_IIb_β_3_ integrin [25]. Therefore, we evaluated whether TQ5 interrupts platelet aggregation via directly binding to the platelet α_IIb_β_3_ integrin. The observed fluorescence intensity revealed that 2 µg/mL FITC-triflavin directly bound to platelets and the intensity was potently reduced in the presence of 5 mM EDTA (negative control) (Figure 2D). However, FITC-triflavin binding to the α_IIb_β_3_ integrin was not affected by TQ5 at 3 and 5 µM, indicating that TQ5 does not directly bind to the platelet α_IIb_β_3_ integrin.

#### 2.1.5. TQ5 on Cyclic Nucleotides Formation

**Figure 3:**
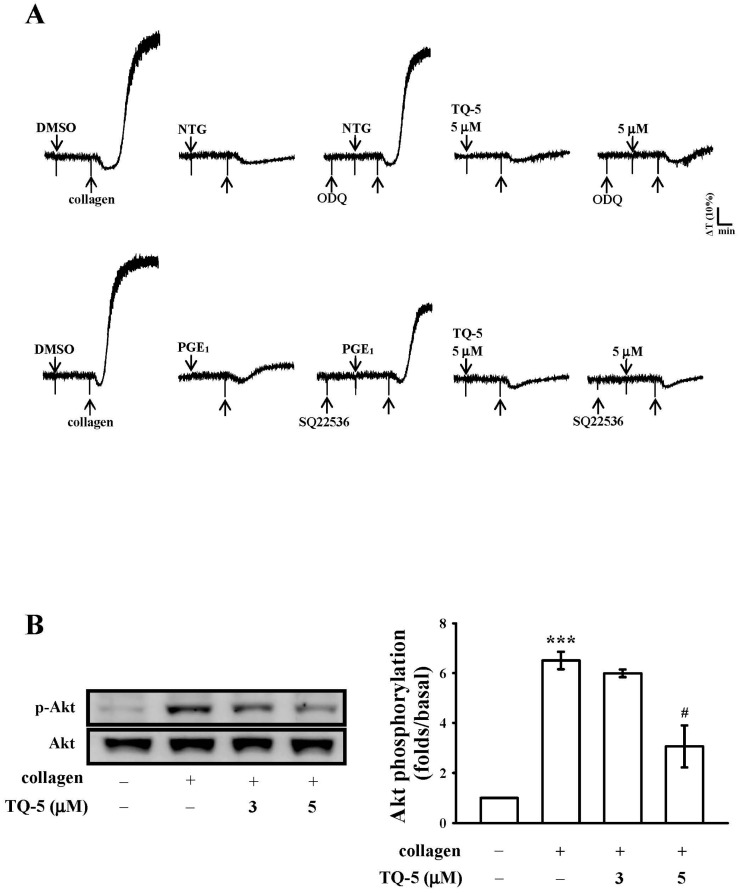
The influence of TQ5 on collagen-induced cyclic nucleotides formation and Akt phosphorylation. (**A**) Washed platelets (3.6 × 10^8^ cells/mL) were preincubated with 10 µM NTG, 0.1 nM PGE1, or 5 µM TQ5 in the absence or presence of 20 µM ODQ or 100 µM SQ22536, and 1 µg/mL collagen was subsequently added to trigger platelet aggregation; (**B**) Platelets (1.2 × 10^9^ cells/mL) were pretreated with 3 and 5 µM TQ5 and 1 µg/mL collagen was subsequently added to induce platelet activation; The cells were collected, and subcellular extracts were analyzed for Akt phosphorylation. Data are presented as the means ± standard error of mean (S.E.M). (*n* = 4). ***p < 0.001 compared with the control group; # *p* < 0.05 compared with the positive control group (collagen only).

As shown in Figure 3A, 10 µM of guanylate cyclase inhibitor ODQ and 100 µM of adenylate cyclase inhibitor SQ22536 significantly reversed the inhibition of collagen-induced platelet aggregation mediated by 10 µM nitroglycerin (NTG) and 1 µM PGE1, respectively. However, neither ODQ nor SQ22536 considerably reversed the inhibition of 5 µM TQ5 mediated collagen-induced platelet aggregation. These results indicated that the mechanism of the TQ5-mediated inhibition of platelet aggregation does not depend on the formation of cyclic nucleotides (e.g., cyclic AMP or cyclic GMP) in human platelets.

#### 2.1.6. TQ5 Attenuated Protein Kinase B (Akt) and c-Jun N-Terminal Kinase (JNK) Phosphorylation in Collagen-Induced Human Platelets

**Figure 4:**
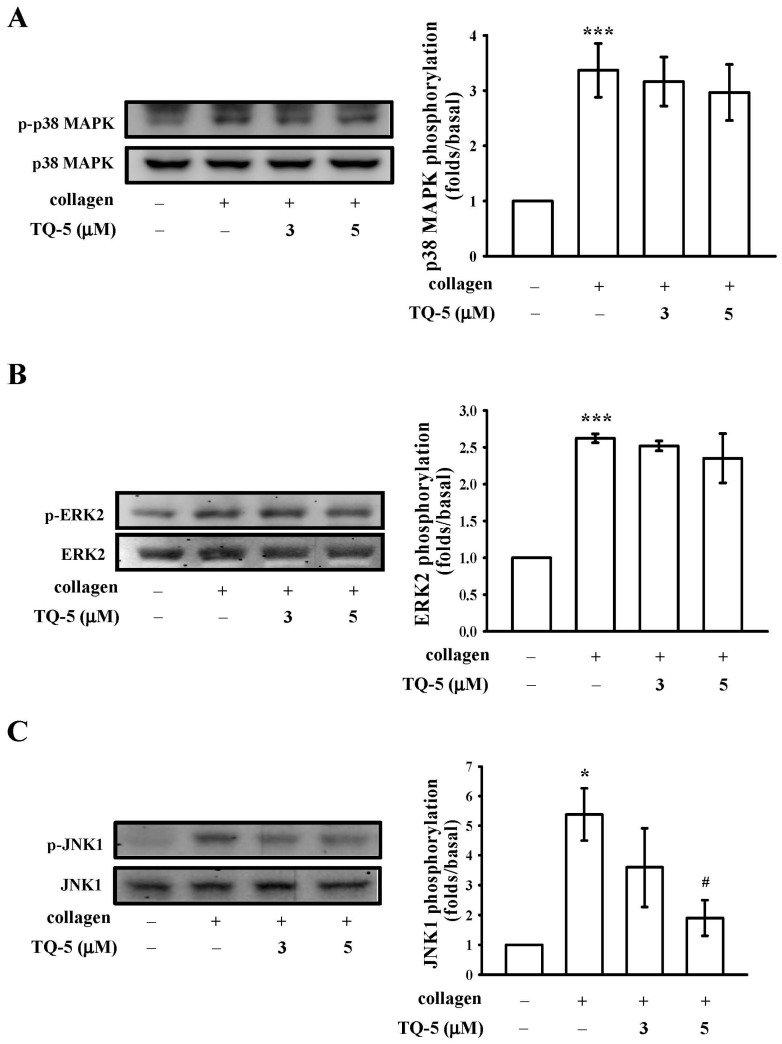
TQ5 on the phosphorylation of MAPK induced by collagen in human platelets. Washed platelets (1.2 × 10^9^ cells/mL) were incubated with solvent control (0.5% DMSO) or TQ5 (3 and 5 µM) and then treated with 1 µg/mL collagen to induce platelet activation. The subcellular extracts were analyzed for the phosphorylation of p38 MAPK (**A**), ERK2 (**B**) and JNK1 (**C**) by western blotting. Data are presented as the mean ± S.E.M. (*n* = 3). *** *p* < 0.001 and * *p* < 0.05 compared with the solvent control group (resting); # *p* < 0.05 compared with the positive control group (collagen only).

The phosphorylation of mitogen-activated protein kinases, MAPKs (ERK, p38, and JNK) and Akt in platelets are closely associated with platelet activation and aggregation [26]. Hence, we determined whether TQ5 inhibited MAPK and Akt phosphorylation in collagen-stimulated platelets. As shown in Figure 4A,B although collagen-induced p38MAPK and ERK phosphorylation was unaffected by TQ5 (3–5 µM), Akt and JNK phosphorylation was dose-dependently suppressed by TQ5. Moreover, TQ5 significantly destroyed the phosphorylation of Akt and JNK at its maximum concentration of 5 µM (Figure 3B and Figure 4C). These results infer that Akt/JNK signaling is involved in TQ5’s antiplatelet activity.

#### 2.1.7. Ex Vivo and In Vivo Studies of TQ5 in Antithrombotic Activity

**Figure 5:**
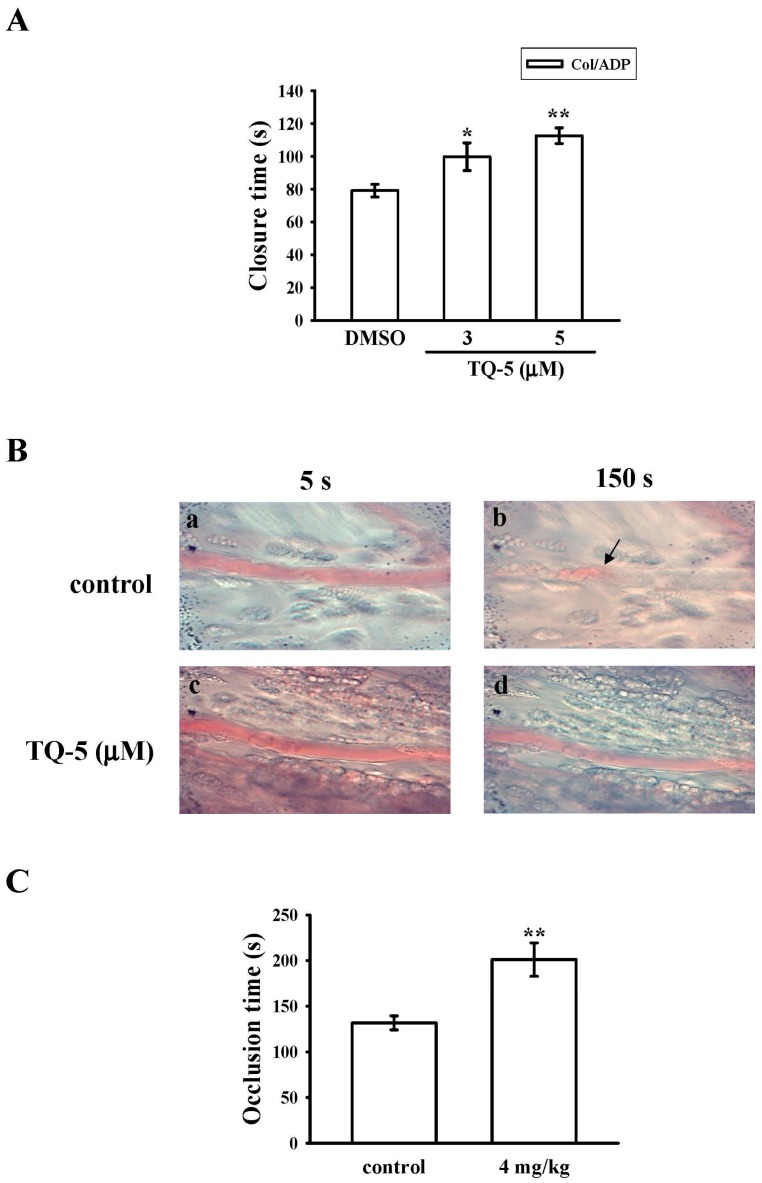
Effects of TQ5 on closure time according to PFA-100 analysis and thrombotic platelet plug formation in the mesenteric venules of mice. (**A**) The shear-induced platelet plug formation in whole blood was determined by CT, as described in the materials and methods section; (**B**) Mice were administered an intravenous bolus of DMSO (control) or TQ5 (4 mg/kg), and the mesenteric venules were irradiated to induce microthrombus formation (occlusion time), as described in the materials and methods section; Microscopic images (magnification ×400) of DMSO-treated controls (**a**,**b**) and the TQ5 (4 mg/kg)-treated groups (**c**,**d**) were recorded 5 s (**a**,**c**) and 150 s (**b**,**d**) after irradiation; (**C**) Bar diagrams are representative examples of six similar experiments. The arrows indicate platelet plug formation. Data ((**A**), *n* = 5 and (**C**), *n* = 6) are presented as the means ±S.E.M. * *p* < 0.05 and ** *p* < 0.01 compared with the control group.

In this study, the shear-induced platelet plug formation in whole blood was tested ex vivo. Platelet function analyser (PFA)-100 instrument was used to mimic the in vivo conditions of blood vessel injury, in which platelets are exposed to a high shear rate. The closure times of collagen-ADP (CADP) in whole-blood control and solvent control [(0.5% dimethyl sulfoxide DMSO)] samples are given in Figure 5A. The treatment of 3 and 5 µM of TQ5 increased the CT of CADP (Figure 5A) in a dose-dependent manner (*p* < 0.05 and *p* < 0.01). Furthermore, we investigated the effect of TQ5 on thrombus formation in vivo. The occlusion time in microvessels pretreated with 15 µg/kg of fluorescein sodium was approximately 150 s. When TQ5 was administered at 4 mg/kg after pretreatment with fluorescein sodium, the occlusion times were significantly prolonged compared with those of DMSO-treated controls (Figure 5B,C).

### 2.2. Discussion

The activation of coagulation cascade and increased platelet aggregation are associated in thrombotic events of cancer patients [27]. Chemotherapeutics may extend this effect and stimulate vascular thromboembolic events (VTEs) by worsening endothelial damage, enhancing platelet aggregation, and increasing oxidative damage leading to vascular toxicity [28]. Among platinum-based chemotherapy agents, cisplatin is reported to have a high incidence of treatment-related VTE [29]. Gemcitabine, in combination with a platinum-agent, has been associated with increased thrombotic and vascular side effects [30,31]. Therefore, a new metal-based inhibition of coagulation and platelet aggregation are required to develop the range of curable vascular diseases, reduce toxic side effects, and overcome platinum resistance. In the present study, we found a newly synthesized ruthenium metal complex, TQ5, potentially inhibits platelet aggregation stimulated by collagen. The observed effect against collagen is more potent than that of a well-established antithrombotic aspirin. The results of this study also show that the Akt and JNK pathway mainly contributed to the inhibition of collagen-induced platelet aggregation, ATP release, and [Ca^2+^]_i_ mobilization by TQ5.

Numerous aggregation-inducing molecules, such as Ca^2+^, thromboxane TxA2, etc., are commonly generated by thrombin, collagen, and ADP. TxA_2_ produces IP_3_ to mobilize [Ca^2+^]_i_ through the G-protein-coupled receptor/PLC-β pathway, and constricts the blood vessel tract [32], which enforces thrombus formation. Many agonists such as collagen, thrombin, and ADP mobilize [Ca^2+^]_i_ to phosphorylate the Ca^2+^/calmodulin-dependent myosin light chain (20 kDa), which plays a role in the secretion of granules such as serotonin and ATP [33], and platelet aggregation. Therefore, the inhibition of [Ca^2+^]_i_ mobilization and ATP production are very important for evaluating the antiplatelet effect of a substance. As anticipated, in this study, TQ5 potently inhibited collagen-induced [Ca^2+^]_i_ mobilization and ATP production in human platelets, indicating that TQ5 inhibits platelet aggregation through suppressing [Ca^2+^]_i_ mobilization and ATP production. It is thought that the inhibition of ATP secretion by TQ5 results from the elevation of the Ca^2+^-antagonistic molecule cAMP and the subsequent inhibition of [Ca^2+^]_i_ mobilization. Human platelet activation is inhibited through intracellular cyclic AMP and cyclic GMP-mediated pathways, and the importance of cyclic nucleotides in modulating platelet activation is firmly established [34]. Elevated levels of cyclic nucleotides inhibit most platelet responses and reduce [Ca^2+^]_i_ through Ca^2+^ uptake by the dense tubular system, which suppresses the activation of PLC and PKC [29]. Thus, cyclic AMP and cyclic GMP act synergistically to inhibit platelet activation. Here, it is observed that neither SQ22536 nor ODQ significantly reversed the TQ5-mediated inhibition of collagen-induced platelet aggregation, and 5 µM of TQ5 had no effects on cyclic AMP or cyclic GMP in human platelets. Therefore, this indicates that the TQ5-mediated inhibition of platelet activation is not dependent on intracellular cyclic nucleotide formation. This result is in accordance with our recent study, in which we found that nobiletin, a bioactive polymethoxylated flavone isolated from citrus fruits, inhibited collagen-induced platelet aggregation, without affecting intracellular cyclic nucleotide formation [35].

The PI3K-Akt pathway has been revealed to support platelet activation by GPVI through the regulation of the serine/threonine kinase Akt [36]. Mouse and human platelets express all three known isoforms of Akt, i.e., Akt1, Akt2, and Akt3, and all have been shown to contribute to platelet activation [37]. Akt family proteins are regulated through the phosphorylation of Thr308 and Ser473 by phosphoinositide-dependent kinase 1 (PDK1) and the mammalian target of rapamycin complex 2, respectively [38]. The generation of phosphatidylinositol 3,4,5-trisphosphate by PI3K recruits PDK1 and Akt to the plasma membrane via their pleckstrin homology (PH) domains, leading to the PDK1-dependent phosphorylation of Akt. In addition, Akt can be phosphorylated by protein kinase C (PKC) and by Ca^2+^/calmodulin-dependent protein kinase kinase (CaMKK), independently of PI3K [39]. Activation by collagen is found to be impaired in mouse platelets deficient in Akt1 [40]. In this study, it has been shown that TQ5 significantly inhibited collagen-induced Akt phosphorylation, but it did not effectively obstruct the phosphorylation of PLCγ2/PKC (data not shown), proposing that the TQ5-potentiated inhibition of platelet activation involves the inhibition of Akt signaling pathways.

Studies with inhibitors and/or genetic manipulations have demonstrated that MAPKs greatly contribute to the platelet responses of various agonists [41]. MAPKs are divided into four subgroups: ERK, JNK, big mitogen-activated protein kinase 1 (BMK1; ERK5), and p38. Among these, ERK, JNK, and p38 are expressed in platelets, and are regulated by a wide range of receptors. Studies have explained that various growth factors and hormone-induced cellular proliferation can activate the ERK1/2 signaling pathway via a Ras/Raf1/MEK1 signaling cascade [42]. Additionally, various inflammatory cytokines and stress stimuli that lead to cellular apoptosis activate JNK1/2 and p38MAPK [42]. The pathophysiological roles of JNK1/2 and ERK1/2 in platelets are unclear, but evidence suggests that the suppression of α_IIb_β_3_ integrin activation or platelet activation may be involved [43]. On the other hand, previous studies have presented and demonstrated that JNK^−/−^ platelets are associated with an increased bleeding time, decreased integrin α_IIb_β_3_ activation, and severe granule secretion impairment [42]. Therefore, it seems that the inhibition of JNK phosphorylation plays an important role in the platelet activation process. Consistent with this spectacle, we found that TQ5 markedly inhibited collagen-induced JNK phosphorylation.

It has been noted that ruthenium complexes have been found as an attractive alternate for platinum due to several favorable properties suited to rational anticancer drug design and biological applications [44,45]. Several studies have proposed that antiplatelet therapy could be beneficial for cancer treatment [46]. Thus, in this study, the observed antiplatelet effect of the novel ruthenium metal complex, TQ5, may provide evidence that this compound could be one of the most promising anticancer agents. However, further studies will be required to examine the TQ5’s antiplatelet effect on cancer treatment.

## 3. Experimental Section

### 3.1. Materials

Collagen (type I), 9,11-dideoxy-11α,9α-epoxymethano prostaglandin (U46619), luciferin-luciferase, thrombin, SQ22536, phorbol-12,13-dibutyrate (PDBu), and 1*H*-[1,2,4]qxadiazolo[4,3-a]quinoxalin-1-one (ODQ) were purchased from Sigma (St. Louis, MO, USA). The anti-phospho-c-Jun N-terminal kinase (JNK) (Thr^183^/Tyr^185^), anti-phospho-p38 mitogen-activated protein kinase (MAPK) monoclonal antibodies (mAbs), and the anti-phospho-p44/p42 extracellular signal-regulated kinase (ERK) (Thr^202^/Tyr^204^) were purchased from Cell Signaling (Beverly, MA, USA). The anti-phospho-p38 MAPK Ser^182^ mAb was purchased from Santa Cruz (Santa Cruz, CA, USA). The anti-phospho-Akt (Ser^473^) and anti-Akt mAbs were purchased from Biovision (Mountain View, CA, USA). The horseradish peroxidase (HRP) conjugated donkey anti-rabbit immunoglobulin G (IgG), the Hybond-P polyvinylidene difluoride (PVDF) membrane, the sheep anti-mouse IgG, and the enhanced chemiluminescence western blotting detection reagent were purchased from Amersham (Buckinghamshire, UK). The TQ5 was dissolved in DMSO and stored at 4 °C.

### 3.2. Synthesis of Ligand 4-Phenyl-2-pyridin-2-yl-quinazoline (L)

2-aminobenzophenone (1.97 g, 10 mmol) and 2-pyridinecarboxaldehyde (1.07 g, 10 mmol), ammonium acetate (2.3 g, 30 mmol), and iodine (0.05 g, 0.2 mmol) were added to a 100 mL round-bottom flask in 30 mL ethanol. The resulting mixture was heated at 50 °C for 5 h. The solvent was evaporated on a rotary evaporator and the resulting solid was washed with water and extracted from CHCl_3_. The combined organic phases were dried over anhydrous sodium sulfate (Na_2_SO_4_) and concentrated under vacuum. The residue was purified by column chromatography on silica gel with petroleum ether/ethyl acetate (2:1) as eluent to give ligand (L) (Figure 1A). Yellow solid (1.6 g), Yield 56%; melting point (mp)162–168 °C; ^1^H NMR (400 MHz, CDCl_3_) δ 8.93–8.92 (d, 1H, *J* = 4 Hz), 8.78–8.76 (d, 1H, *J* = 8 Hz), 8.39–8.36 (d, 1H, *J* = 12 Hz), 8.17–8.15 (d, 1H, *J* = 8 Hz), 7.95–7.88 (m, 4H), 7.63–7.59 (m, 4H), 7.43–7.40 (t, 1H, *J* = 6 Hz); ^13^CNMR (400 MHz, CDCl_3_) δ 168.8, 159.1, 155.4, 151.8, 150.2, 137.3, 136.9, 133.7, 130.4, 129.9, 128.7, 127.8, 127.0, 124.5, 124.3, 122.1; Anal. found (calcd) for C_19_H_13_N_3_: C, 80.50 (80.54); H, 4.58 (4.62); N, 14.85 (14.83). ESI-MS *m*/*z* 284 [M + H]^+^; HRMS (ESI) calcd for C_19_H_14_N_3_: 284.1188 [M + H]^+^, Found: 284.1182 [M + H]^+^.

### 3.3. Synthesis of [Ru(η^6^-Cymene)(L)Cl]BF_4_ (TQ5)

[Ru(*p*-cymene)(Cl)_2_]_2_ (0.12 g, 0.2 mmol) and L (0.11 g, 0.4 mmol) were suspended in methanol (20 mL) and stirred at room temperature for 2 h. Upon the addition of ammonium tetrafluoroborate (0.2 g, 0.60 mmol), the yellow solution changed to an orange coloured solution. After 24 h, the solution was evaporated and the obtained solid was washed with dichloromethane and filtered off. The residue was washed with diethyl ether (40 mL) and dried under vacuum. The desired products were recrystallized from dichloromethane: hexane mixture to give orange coloured microcrystals (0.22 g). Yeild 87%; mp 223–225 °C; ^1^H NMR (400 MHz, CDCl_3_) δ 9.50–9.49 (d, 1H, *J* = 4 Hz), 9.35–9.34 (d, 1H, *J* = 4 Hz), 8.70–8.67 (t, 2H, *J* = 6 Hz), 8.29–8.18 (m, 2H), 8.11–8.05 (m, 2H), 7.93–7.87 (m, 2H), 7.77–7.75 (d, 2H, *J* = 8 Hz), 7.71–7.60 (m, 1H), 6.09–6.08 (d, 1H, *J* = 4 Hz), 5.97–5.95 (d, 1H, *J* = 8 Hz), 5.86–5.84 (d, 1H, *J* = 8 Hz), 5.30 (s, 1H), 2.40–2.35 (m, 1H), 2.26 (s, 3H), 0.92–0.82 (m, 6H); ^13^C-NMR (400 MHz, DMSO-*d*_6_) δ 175.1, 169.8, 156.4, 152.9, 148.5, 140.5, 137.7, 136.7, 134.7, 131.2, 130.8, 129.28, 128.7, 125.8, 123.9, 122.5, 104.7, 104.4, 87.1, 83.4, 30.3, 21.6, 18.3; Anal. found (calcd) for C_29_H_27_N_3_: C, 54.40 (54.35); H, 4.21 (4.25); N, 6.53 (6.56); ESI-MS: *m*/*z* = 553 [M − BF_4_]^+^.

### 3.4. Platelet Aggregation and ATP Release

The methods described by Sheu et al. [47] and Lin et al. [48] were followed for the preparation of the human platelet suspensions. Blood was collected from healthy human volunteers who did not take medication during the preceding two weeks and was mixed with acid-citrate-dextrose solution (1:9). The blood samples were subjected to centrifugation at 120× *g* for 10 min, and platelet-rich plasma (PRP) was collected. PRP was supplemented with PGE_1_ (0.5 µM) and heparin (6.4 IU/mL), and was then incubated for 10 min at 37 °C. After centrifugation at 500× *g* for 10 min, the platelet pellets were suspended in Tyrode’s solution containing 3.5 mg/mL bovine serum albumin (BSA), pH 7.35 (NaCl 137 mM, KCl 2.7 mM, MgCl_2_ 1 mM, NaH_2_PO_4_ 0.2 mM, NaHCO_3_ 12 mM, and glucose 5.5 mM). Then, PGE_1_ (0.5 µM), apyrase (1.0 U/mL), and heparin (6.4 IU/mL) were added, and the mixture was incubated for 10 min at 37 °C. The mixtures were centrifuged at 500× *g* for 10 min and subjected for the repeated washing procedure. Finally, the platelet pellets were resuspended by Tyrode’s solution, and then CaCl_2_ was added to platelet suspensions in which the concentration of Ca^2+^ was 1 mM. This study was approved by the Institutional Review Board of Taipei Medical University and conformed to the directives of the Helsinki Declaration.

As previously described [47,48], platelet aggregation was measured according to the turbidity of platelet suspensions and recorded by a Lumi-Aggregometer (Payton Associates, Scarborough, ON, Canada). Before the addition of agonists to induce platelet aggregation, the platelet suspensions (3.6 × 10^8^ cells/mL) were pretreated with various concentrations of TQ5 or an isovolumetric solvent control (0.5% DMSO) for 3 min. A light-transmission unit was used to present the extent of platelet aggregation. For the measurement of ATP release, 20 µL of luciferin-luciferase mixture was added 1 min before adding collagen, and the relative amount of ATP release was compared to the solvent control.

### 3.5. Measurement of Relative Ca^2+^ Mobilization by Fura 2-AM Fluorescence

Citrated whole blood was centrifuged at 120× *g* for 10 min. The supernatant was incubated with Fura 2-AM (5 µM) for 1 h. Human platelets were then prepared as described above. Finally, the external Ca^2+^ concentration of the platelet suspensions was adjusted to 1 mM. The relative Ca^2+^ mobilization was measured as described previously [49].

### 3.6. Immunoblotting

Washed platelets (1.2 × 10^9^ cells/mL) were pre-incubated with 3 and 5 µM TQ5 or 0.5% DMSO for 3 min. The reaction was stopped, and the platelets were immediately re-suspended in 200 µL of a lysis buffer. Samples containing 80 µg of protein were separated on a 12% acrylamide gel using sodium dodecylsulfate polyacrylamide gel electrophoresis (SDS-PAGE), and the proteins were electrotransferred to the PVDF membranes by using a Bio-Rad semidry transfer unit (Hercules, CA, USA). Blots were blocked with TBST (10 mM Tris-base, 100 mM NaCl, and 0.01% Tween 20) containing 5% BSA for 1 h and probed with various primary antibodies. The membranes were incubated with the HRP-linked anti-mouse IgG or anti-rabbit IgG (diluted 1:3000 in TBST) for 1 h. Immunoreactive bands were detected using an enhanced chemiluminescence system. Ratios of the semiquantitative results were obtained by scanning the reactive bands and quantifying the optical density by using a video densitometer and Bio-profil Biolight software, Version V2000.01 (Vilber Lourmat, Marne-la-Vallée, France).

### 3.7. Analysis of Platelet Function in Whole Blood

A Dade Behring PFA-100 System (Siemens Healthcare, Marburg, Germany) was used to measure platelet function [25]. Cartridges containing a collagen-ADP (CADP)-coated membrane were preincubated with DMSO for 2 min. Aliquots of whole blood (0.8 mL/cartridge) were applied to the cartridges before the contents were exposed to high-shear-flow conditions (5000–6000/s). The closure time (CT) was defined as the time required for the platelet plug to occlude the aperture in the membrane [25]. Finally, 3 and 5 mg/kg TQ5 was administered to measure the closure time.

### 3.8. Fluorescein-Induced Thrombus Formation in the Microvessels of Mouse Mesentery

The protocols conformed to the Guide for the Care and Use of Laboratory Animals (NIH publication No. 85–23, 1996). The method to measure thrombus formation was performed as described previously [48,49]. In brief, an external jugular vein was cannulated with a PE-10 to intravenously administer the dye and drugs after mice were anesthetized. Venules (30–40 µm) were selected under a microscope. After administering 15 µg/kg sodium fluorescein, the selected venules were irradiated at wavelengths below 520 nm to produce a microthrombus, and the time required to occlude the microvessel as a result of thrombus formation (occlusion time) was recorded. Then, 4 mg/kg TQ5 was administered to evaluate its antithrombotic effects.

### 3.9. Statistical Analysis

The experimental results are expressed as the means ±S.E.M., and are accompanied by the number of observations (*n*). Values of n refer to the number of experiments, and each experiment was conducted using different blood donors. The paired Student’s *t*-test was used to determine significant differences in the occlusion time in mice. Differences between groups in other experiments were assessed using an analysis of variance. When this analysis indicated significant differences among group means, the groups were compared using the Student–Newman–Keuls method. Pb.05 indicated statistical significance. Statistical analyses were performed using SAS Version 9.2 (SAS Inc., Cary, NC, USA).

## 4. Conclusions

In this study, we report that the TQ5 potentially suppressed platelet aggregation in vitro and thrombotic plug formation in vivo. The underlying molecular mechanisms of the inhibitory effects of TQ5 on platelet function seem to be the suppression of Akt/JNK signaling cascades. These alterations reduce the level of ATP and [Ca^2+^]_I_, and ultimately inhibit platelet aggregation. Overall, our data suggest that TQ5 may be considered as a potent therapeutic agent against abnormal platelet activation-related diseases such as thrombosis and arteriosclerosis.

## Supplementary Materials

Supplementary materials can be found at www.mdpi.com/1422-0067/18/5/916/s1.
