# Supplementary material for: Antiplatelet Activity of a Newly Synthesized Novel Ruthenium (II): A Potential Role for Akt/JNK Signaling
Source: Int J Mol Sci. 2017 Apr 27;18(5):916. doi: 10.3390/ijms18050916 (PMC5454829; doi:10.3390/ijms18050916)
Supplement: Supplementary file 1 [file ijms-18-00916-s001.pdf]

Supporting Information

Antiplatelet activity of a newly synthesized novel ruthenium (II) complex: potential role for Akt/JNK signaling

Themmila Khamrang[1,2*], Kuo-Chen Hung[3*], Chih-Hsuan Hsia[2], Cheng-Ying Hsieh[2], Marappan Velusamy[1] Thanasekaran Jayakumar[2]and Joen-Rong Sheu[2]

[1]Department of Chemistry, North Eastern Hill University, Shillong 793022, India; E-Mail: themmilakhamrang@gmail.com (T.K); mvelusamy@gmail.com (M.V)

[2]Graduate Institute of Medical Sciences and Department of Pharmacology, College of Medicine, Taipei Medical University, Taipei, Taiwan; E-Mail: d119102013@tmu.edu.tw (C.-H.H); hsiehcy@tmu.edu.tw (C.-Y.H); tjaya_2002@yahoo.co.in (T.J); sheujr@tmu.edu.tw (J.-S.R)

[3]Gastroenterologic Surgery Division, Department of Surgery, Yuan's General Hospital, Kaohsiung 249, Taiwan; E-Mail: hcc4723@gmail.com (K.-C.H)

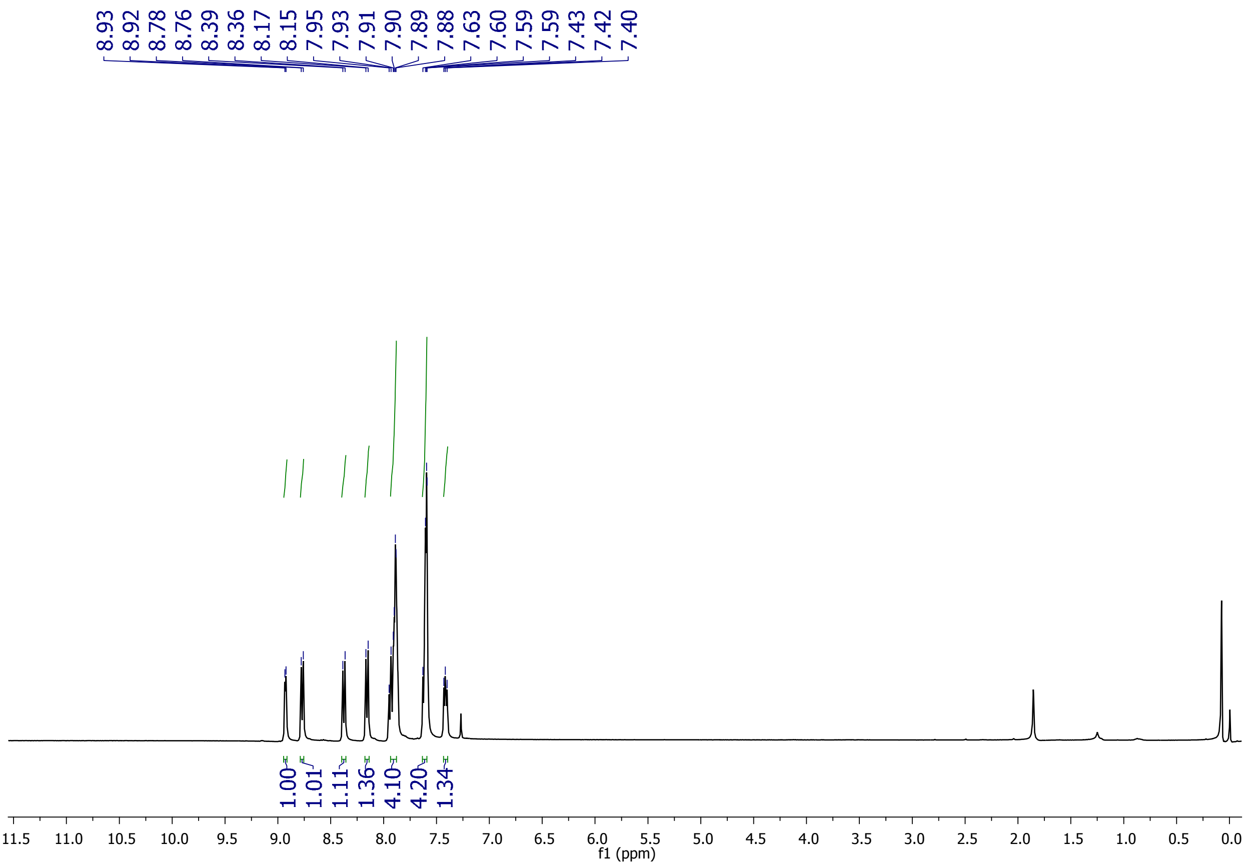

Figure S1: ^{1}H NMR spectrum of **L** in $CDCl_3$

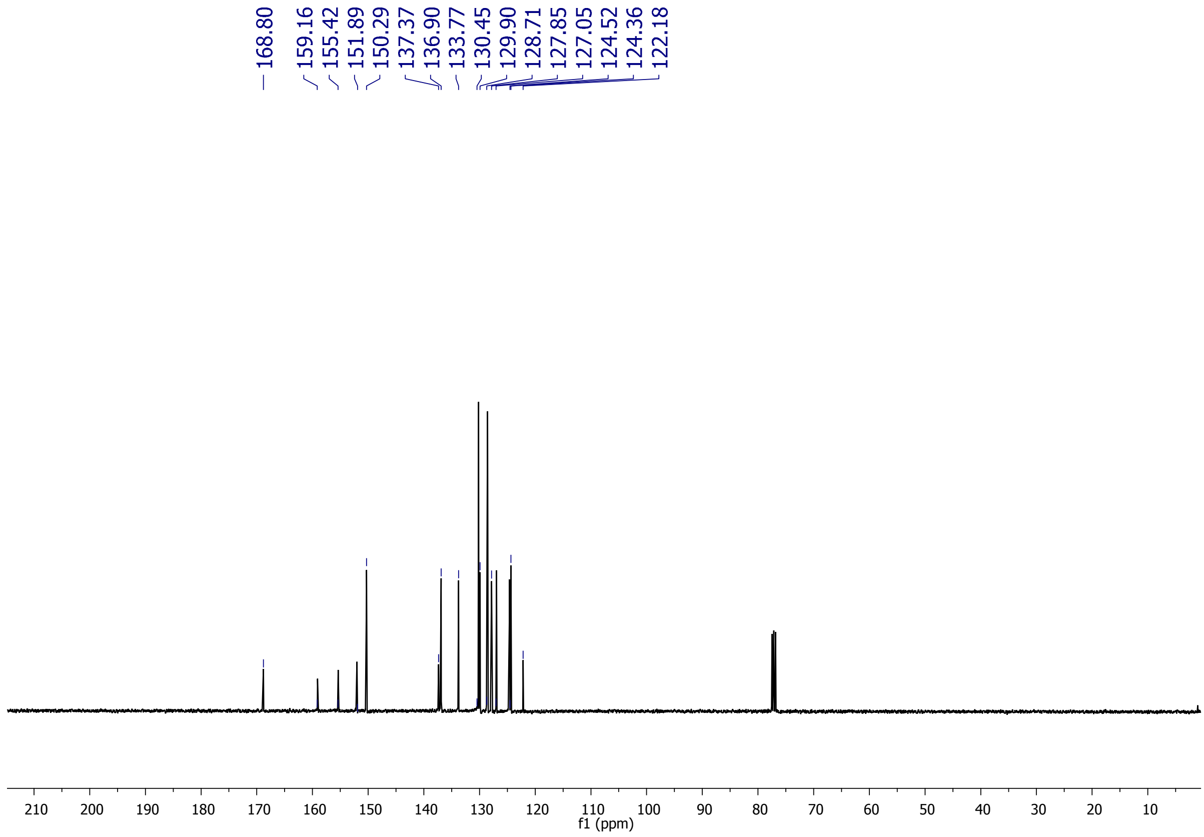

Figure S2: ^{13}C NMR spectrum of **L** in $CDCl_3$

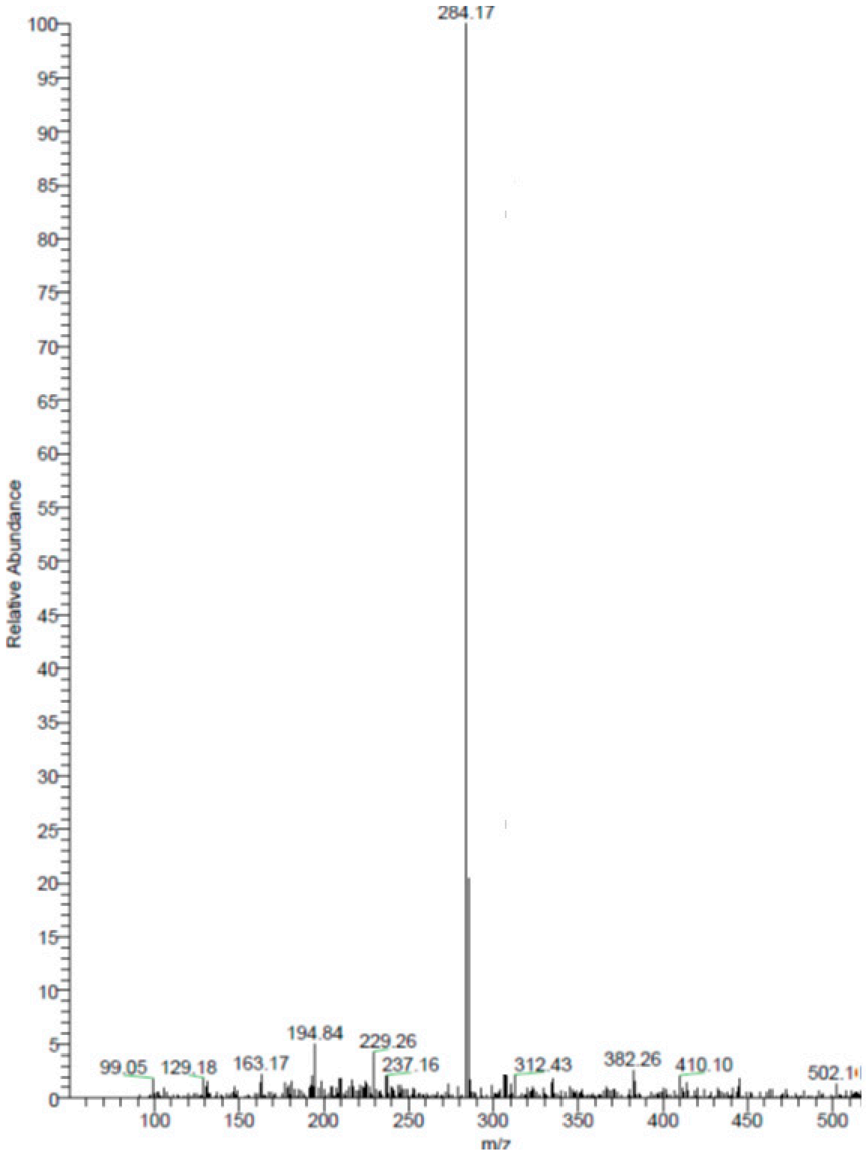

Figure S3: ESI-MS spectrum of **L**.

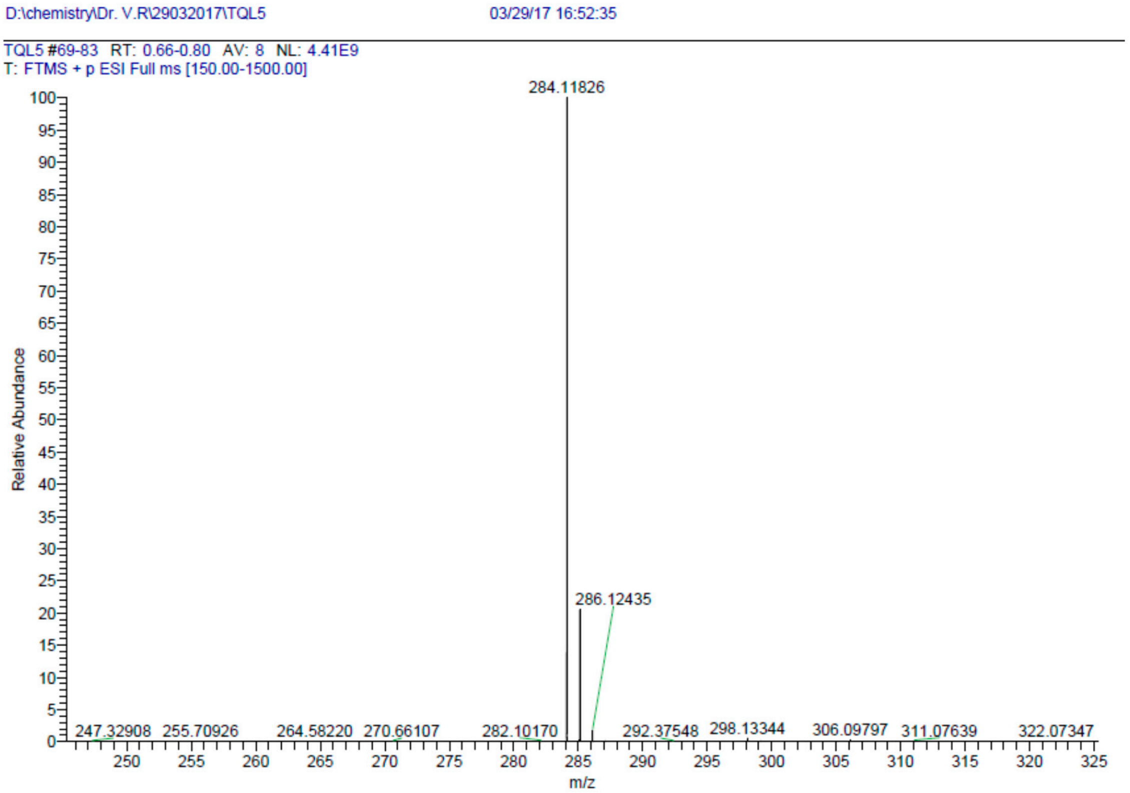

Figure S4. HRMS-ESI for **L**, Calcd for $C_{19}H_{14}N_3$: 284.1188 $[M+H]^+$, Found: 284.1182 $[M+H]^+$.

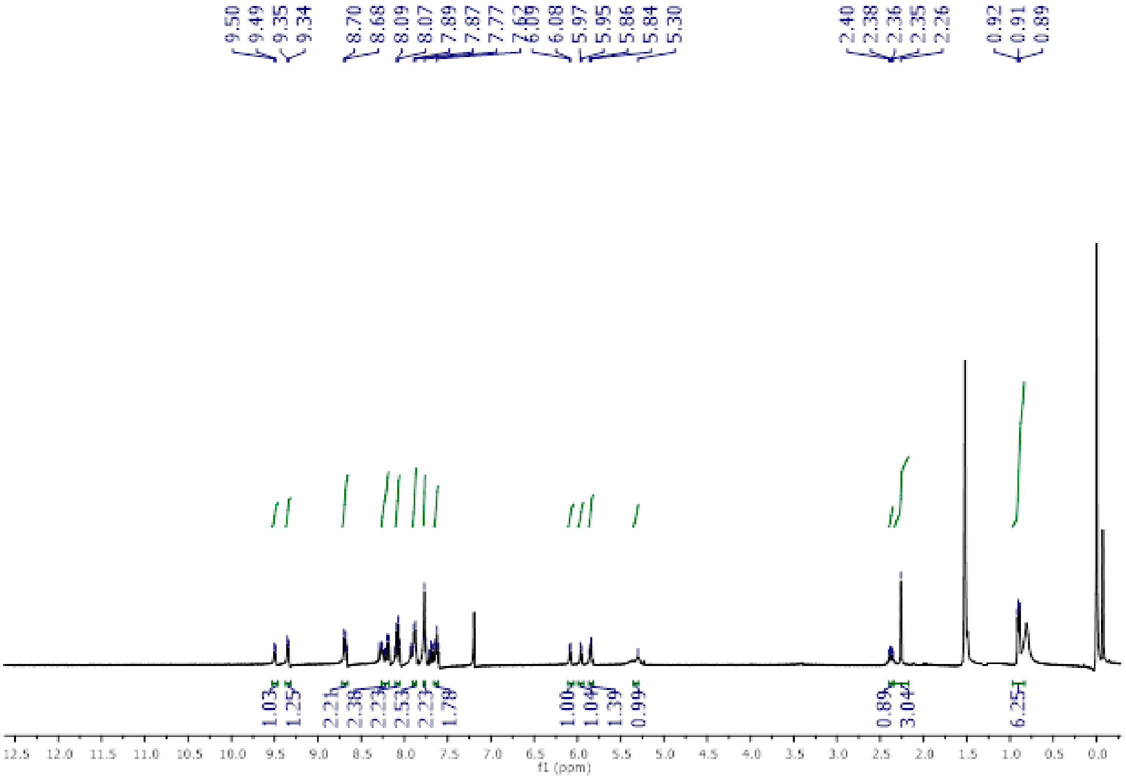

Figure S5: ^{1}H NMR spectrum of TQ-5 Complex in $CDCl_3$

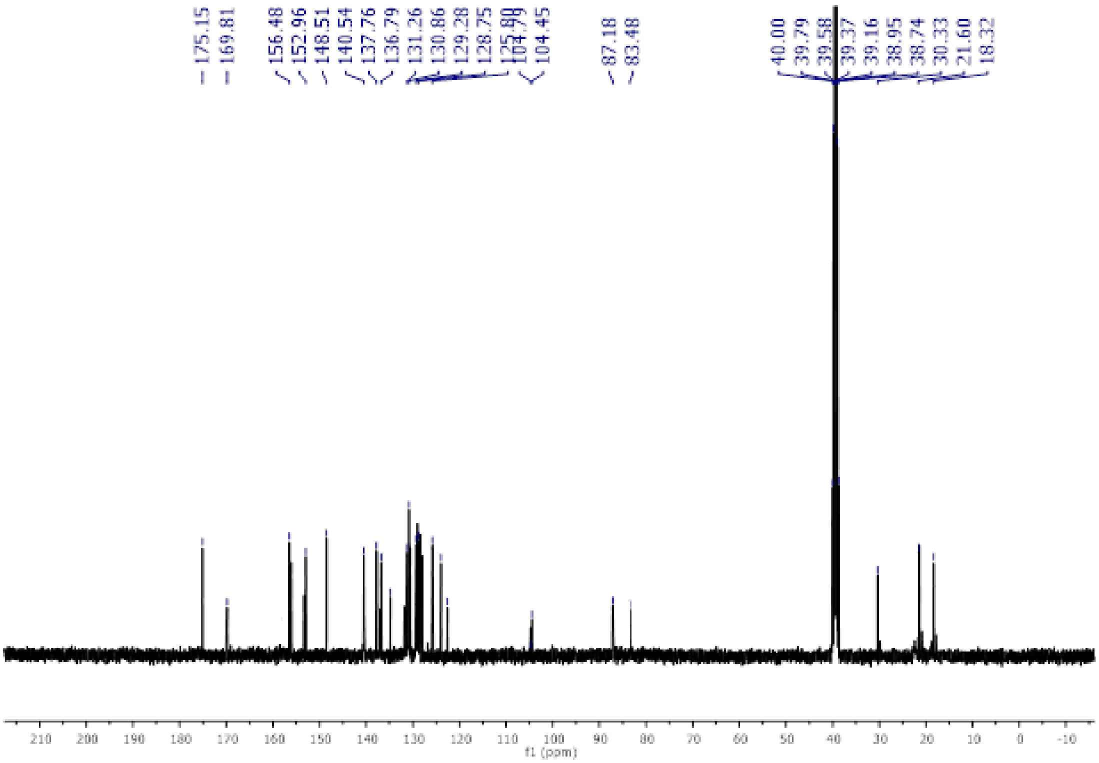

Figure S6: ^{13}C NMR spectrum of TQ-5cmplex in DMSO-d_6

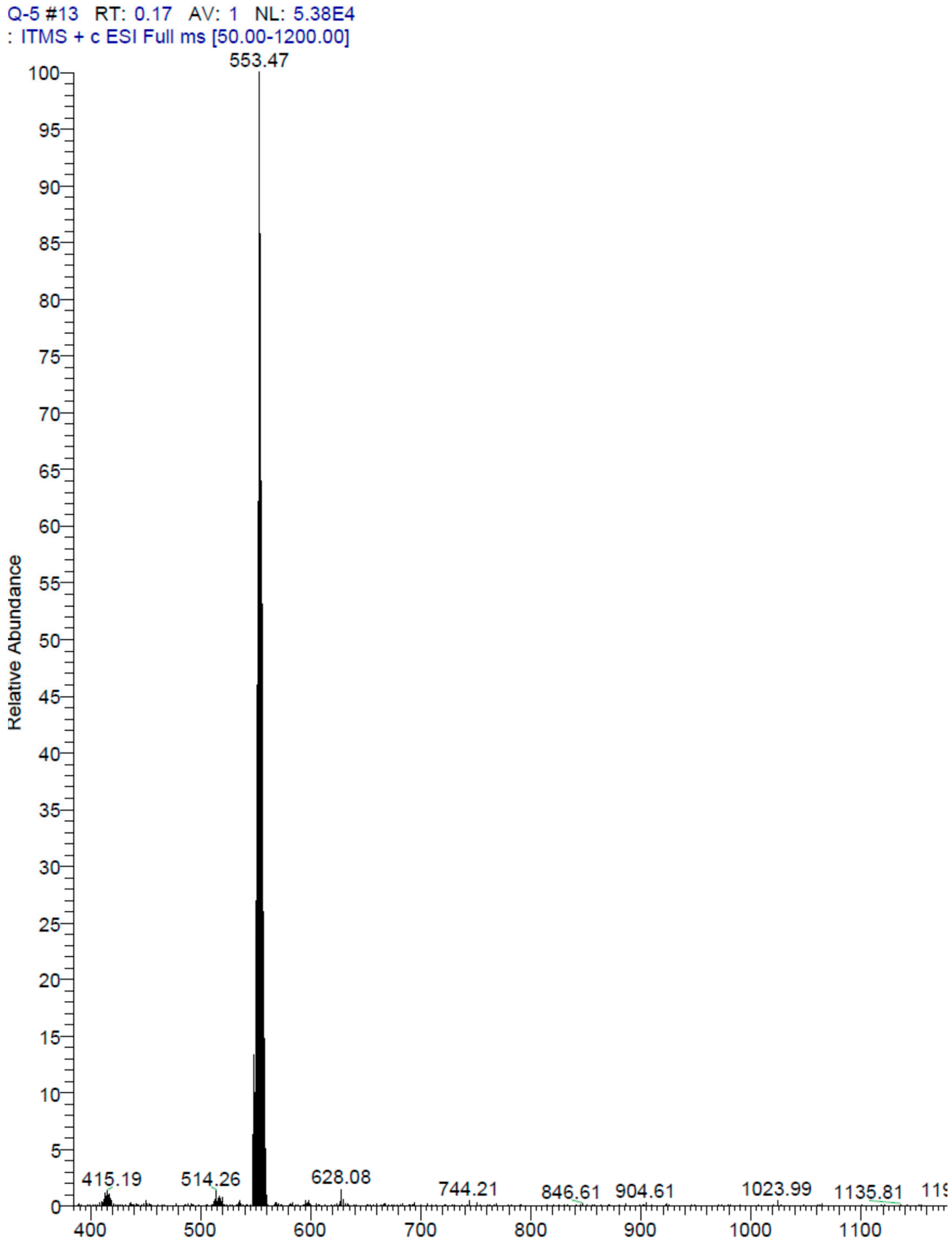

Figure S7: ESI-MS spectrum of TQ-5 Complex.
